# Exploring options for family medicine subspecialisation in South Africa: A proposed way forward following a national workshop

**DOI:** 10.4102/jcmsa.v3i1.258

**Published:** 2025-12-20

**Authors:** Mergan Naidoo, Klaus von Pressentin, Madeleine Muller, Olufemi Omole, Kimera T. Suthiram, Laurel Baldwin-Ragaven

**Affiliations:** 1 Department of Family Medicine, College of Health Sciences, University of KwaZulu-Natal, Durban, South Africa; 2 Department of Family, Community and Emergency Care, Faculty of Health Sciences, University of Cape Town, Cape Town, South Africa; 3 Department of Family Medicine, Faculty of Medicine, Walter Sisulu University, Mthatha, South Africa; 4 Department of Family Medicine, Faculty of Health Sciences, University of the Witwatersrand, Johannesburg, South Africa

**Keywords:** family medicine, subspecialisation specialty areas, national workshop, postgraduate training

## Abstract

Family medicine (FM) is a clinical discipline that provides comprehensive, continuous, contextualised, first contact and person-centred health care. The practice of FM is not limited by the patient’s age, gender or diagnosis, thus encompassing the broad scope of general medical practice. Given the current epidemiological landscape in South Africa, however, there is an increasing demand in primary healthcare spaces (district hospitals, community health centres, and clinics) for in-depth knowledge and advanced skills to respond to the burgeoning complex needs of the population. The potential benefits of FM subspecialty training in relevant areas are therefore significant, including broadening access to expertise and skills for patients and improving career pathing and job satisfaction for Family Physicians (FPs).

Subspecialisation in FM can enhance the quality of clinical care by strengthening the district health system to deliver effectively on the nation’s health priorities. Recognising this potential, the College of FPs (South Africa) set up a task team to explore subspecialisation in FM through a workshop at the 25th Annual South African Academy of FPs in August 2023. This report details the background, proceedings and significant findings of the workshop. It suggests a way forward, including a Delphi study to explore subspecialisation possibilities further and build consensus within the discipline and with other key stakeholders.

**Contribution:**

This article reports on a national workshop convened by the College of FPs of South Africa to explore future subspecialisation pathways in FM. It represents an initial, exploratory phase of a broader research and policy development process rather than a hypothesis-driven empirical study. The purpose of sharing these findings is to document key deliberations, generate dialogue within the Colleges of Medicine, and inform the design of subsequent consensus-building steps, including a Delphi study.

## Introduction

The future of family medicine (FM) in South Africa has been re-imagined at different times over the past several decades.^1^ At various stages in our discipline’s history, some questioned why FM should become a designated speciality at all,^2,3^ given its focus on generalism. Others believed that narrowing the discipline even further was required to meet the country’s needs for specialised medical skills, especially in district hospitals and rural areas.^4^ In 2007, the Health Professions Council of South Africa (HPCSA) recognised FM as a speciality, with the first registrars completing a full-time 4-year registrar training programme in 2011. Subsequently, the FM leadership issued position papers in 2014 and 2022, articulating clear visions for purpose and growth.^5^ The family physician (FP) role is further outlined in the National Development Plan for South Africa, where it is inextricably linked to promoting health through clinical governance and improving the quality of district health services. Currently, FPs are utilised as multipurpose specialists in various settings, including primary health care (PHC) clinics, community health centres, district hospitals, universities and ward-based outreach teams in rural and urban settings. This scope of practice also includes non-clinical responsibilities, such as leadership, clinical and corporate governance, capacity building and research.^5^ FPs thus adapt to the contextual needs of their patients and communities^4^ and are slated to be critical to the rollout of Universal Health Coverage (UHC) in South Africa.^6^

Conversations around FM subspecialisation are happening globally.^7,8^ Yet, there remains a tension between FPs retaining their generalist identity versus becoming more specialised in areas relevant to their interests or the contextual needs of a defined patient population. Subspecialisation may cause the practitioner to lose the breadth of FM competencies attained during training. Reasons for choosing subspecialty training include innovation, research and striving to be the best in their field, while balancing lifestyle considerations such as working hours. Although some countries have already committed to offering additional training in specific subspecialisation categories within FM, there are essential differences in nomenclature and status regarding professional registration, scope of practice and billable services. Table 1^9,10,11,12^ outlines additional specialised training available for FPs after acquiring the primary speciality in several different countries, including South Africa.

**TABLE 1:** Global family medicine subspecialisation training and speciality areas of interest.

Country	Area of expertise	Duration of additional training
**United States**		
Combined residency programmes^9^	FM – Emergency medicine	5 years versus 3 years, with dual board certification in both specialities
	FM – Internal medicine	
	FM – Psychiatry	
	FM – Preventive medicine	
	FM – Osteopathic neuromusculoskeletal medicine	
Fellowships	Addiction medicine	12 months after completion of FM residency
	Faculty development	
	Geriatrics	
	Integrative medicine	
	Obstetrics	
	Preventive medicine	
	Research	
	Sports medicine	
Certificates of added qualification	Adolescent medicine	12–24 months post-FM residency training
	Geriatric medicine	
	Hospice and palliative medicine	
	Pain medicine	
	Sleep medicine	
	Sports medicine	
**Canada**		
Enhanced skills programmes^10^		
Category 1 programme Offered nationally by the Canadian College of Family Physicians	Care of the elderly	12 months post-residency training in FM
	Emergency medicine	
	Sport and exercise medicine	
	Palliative medicine	
	Care of the elderly	
Category 2 programmes Offered by individual universities with programme and content that may vary from site to site	Academic FM	6–12 months post-residency training in FM
	Child health	
	Chronic diseases management	
	Enhanced skills – Individualised programme (varies)	
	Hospitalist	
	Obstetrics and women’s health	
	FM oncology	
	Primary care rheumatology	
**United Kingdom**		
General practitioner with an extended role^11^	Teaching or training	12 months following completion of national GP training
	Research	
	Occupational medicine	
	Minor surgery	
	Dermatology	
	Cosmetic procedures	
	Mental health	
	Cardiology	
	Sports medicine	
	Emergency medicine	
	Dermatology	
	Women’s health	
**Australia**		
Speciality areas of interest (up to 37 different fields)^12^	Paediatrics	12 months
	Women’s health	
	Pregnancy and obstetrics	
Speciality areas of interest (up to 37 different fields)^12^	Psychiatry	12 months following completion of national GP training
	Minor surgery	
	Complementary medicine	
	Men’s health	
	Sports medicine	
	Palliative care	
	Geriatrics	
	Rural generalist fellowship	
**South Africa**		
Subspecialty Certificate† in Allergology of the College of Family Physicians of South Africa	Allergology	18 months in a numbered subspeciality training post after completion of either FM or other specialist training (e.g. Paediatrics)

Note: Please see the full reference list of the article, Naidoo M, Von Pressentin K, Muller M, Omole O, Suthiram KT, Baldwin-Ragaven L. Exploring options for family medicine subspecialisation in South Africa: A proposed way forward following a national workshop. J Coll Med S Afr. 2025;3(1), a258. https://doi.org/10.4102/jcmsa.v3i1.258, for more information.

FM, family medicine; GP, general practitioner.

† , This will soon be renamed as a Fellowship in the subspecialty.

## Subspecialty training in South Africa and rationale for the workshop

Medical specialisation and subspecialisation in South Africa are governed within a legal framework, which currently recognises approximately 30 subspecialties as subdivisions of particular specialities.^13^ Several of these subspecialties are multidisciplinary, with prerequisite entry requirements open to individuals trained in multiple primary specialities. For example, the subspecialisation in Allergology, while awarded by the College of Family Physicians (CFP), is a joint offering with many other disciplines within the Colleges of Medicine of South Africa (CMSA).

As a constituent college of the CMSA, the CFP is responsible for the national exit examinations for specialists and subspecialists in FM (with Allergology being the only subspecialty currently offered by the CFP, with other relevant disciplines). Given the growing interest in FM subspecialist training locally and internationally, the CFP sought a national forum to develop a position for the discipline in the South African context. A task team consisting of this manuscript’s authors facilitated a workshop at the 25^th^ Annual National Family Practitioners Congress on 19 August 2023. This workshop was the first step in exploring and gauging the appeal for FM subspecialist training and/or areas of special interest.

## Workshop process

The workshop had 44 participants, including FPs, registrars from various universities and provinces across South Africa, and invited experts from related fields. These included a rehabilitation medicine specialist and a subspecialist in Allergology, both of whom contributed perspectives on existing and emerging models of subspecialty practice.

The overall aim of the workshop was to explore possible pathways for subspecialisation in FM within the South African context, identify areas of overlap with existing subspecialties, and consider how such roles might strengthen the district health system. Participants were encouraged to discuss not only potential academic or training models but also how subspecialist-trained FPs could function at the PHC level. In this regard, participants envisioned FPs with additional subspecialist competencies – such as in geriatrics, palliative care, or rehabilitation – serving as district-level resources, supporting PHC teams through clinical leadership, capacity building, and referral optimisation.

This workshop formed Round 1 of a planned Delphi process, laying the foundation for subsequent national rounds to refine and prioritise proposed subspecialty areas. Table 2 outlines the workshop format, which began with presentations exploring national and global perspectives of FM subspecialisation. This was followed by inputs from experts engaged in established (Allergology), emerging (Palliative Medicine) and potential new FM subspecialties (Physical and Rehabilitation Medicine). Four facilitated breakout groups asked participants to consider different subspecialisation models and report back.

**TABLE 2:** Workshop outline.

Time allocation (min)†	Topic or activity
5	Introduction
15	Global and local perspectives of subspecialisation in FM
3 × 5	Views from experts in subspecialty areas *An established subspecialty*: Allergology *An emerging subspecialty: Palliative Medicine* *A future possibility*: Physical and Rehabilitation Medicine
45	Breakout sessions in four groups: Partnering with other CMSA colleges that already offer *existing subspecialties* relevant to FM, such as Critical Care, Endocrinology, Geriatric Medicine, Infectious Disease, Rheumatology and Developmental Paediatrics. Engaging with *emerging subspecialties at an advanced stage of development*, such as Palliative Medicine. Initiating *new multidisciplinary subspecialties* with other CMSA colleges, such as Pain Medicine, Rehabilitation Medicine, Preventative Medicine, Addiction Medicine, Academic Medicine and Chronic Disease management. Imagining *new FM-specific subspecialties*, such as Rural and Remote Generalism (Primary Care Hospitalist Medicine), Maternal and Child Health (or Community Obstetrics or Community Paediatrics), Women’s Health, Adolescent Medicine, Sexual and Gender Health and Community FM.
20	Feedback from all groups in the plenary
5	Closure and next steps

FM, family medicine; CMSA, colleges of medicine of South Africa.

† , total duration: 1 h 45 min.

## Feedback from the breakout groups

All four groups viewed opportunities for subspecialisation positively, agreeing that FPs should be able to take on a subspecialty or special interest area (see details in Box 1). The groups highlighted the exploration of crosscutting approaches to link allied disciplines or branches of specialisation with the central generalist ethos of FM. Like other subspecialists who usually work in their primary speciality (e.g. Internal Medicine), with 30% of their work in their subspecialty (e.g. Cardiology, Infectious Disease or Palliative Care), FP subspecialisation could similarly adopt this model. During the plenary discussion, the groupwork informed the following suggestions for the CFP:

- The CFP and South African Academy of Family Physicians (SAAFP) leadership should reach out to members to gauge support for pursuing FM subspecialisation in South Africa. In addition to the task team members, 17 workshop participants expressed interest in a possible Delphi process.
- If sufficient broad-based approval exists, the CFP will work with the CMSA and the other relevant disciplines to add FM to specific existing subspecialties. Potential opportunities for partnering include *inter alia*: Critical Care, Endocrinology, Geriatric Medicine, Infectious Diseases, Developmental Paediatrics and Rheumatology.
- Further discussion would be needed, however, to expand the scope of practice of particular subspecialties. For example, the Infectious Disease subspecialty is usually restricted to adults. Yet, FPs care for patients of all ages, so training must accommodate pregnant women and children.
- The newly conceived FM subspecialties highlighted in the workshop are relevant and appropriately aligned to FM practice. Teams of champions with the appropriate skills and interests will be required to develop these further.
- The rollout of subspecialisations should include negotiating training possibilities. Training platforms for FM subspecialties would traverse the health system continuum and be embedded in communities. Growing a learning environment through communities of practice across disciplines and professions should inform blended teaching and learning strategies.
- Funding options, such as the Discovery Foundation awards, could be explored to expand training posts.

BOX 1 Groupwork feedback.

**Table:** 

Groups 1 and 2: Existing and emerging subspecialities	Groups 3 and 4: New multidisciplinary and FM-specific subspecialities
Groups 1 and 2 explored collaboration opportunities with cognate disciplines on mutual importance and overlap. Linking into existing subspecialities would be the easiest way to expand FM-specific pathways for subspecialisation. **These groups highlighted the advantages**: Subspecialisation provides an opportunity for further development in a field of interest, making the profession more attractive and promoting job satisfaction. Another advantage of FM subspecialisation is that our speciality attends to patients across their lifespan. Additional training in almost any subspecialty allows us to apply knowledge and skills to an entire population, regardless of age or state (e.g. pregnancy). Family physicians with subspecialist training would be leaders and return skills to practice settings, such as district hospitals, PHC clinics and the wider community. This approach differs from many other subspecialties in that it ensures access at primary levels of care, not just in tertiary or quaternary settings. For example, an FP with Geriatrics subspecialisation could be optimising elder care within the community; an FP with Developmental Paediatrics would be able to set up neurodevelopmental assessment structures and support for children within their rural sub-district; and, an FP with Critical Care could initiate high-care interventions in larger district hospitals whilst patients await ICU beds centrally. Similarly, this leadership could translate into other innovations, such as setting up palliative care services or holistically managing the diabetic burden in a local community. When fully established, these programmes could be integrated into extended training platforms within the district health services.	Groups 3 and 4 discussed and mapped at least five potential foci for specialisation. The groups then assigned champions to these focus areas. **‘Perinatal health priorities in primary care’:** combined focus on reproductive, maternal and child health. FPs with Obstetrics diplomas may lead this area. **‘Sexual health and gender-affirming healthcare’:** drivers could be colleagues affiliated with the Southern African Sexual Health Association (SASHA) and the Professional Association for Transgender Health South Africa (PATHSA). **‘Community ambulatory care activities’:** combined focus on community and population health, community-oriented primary care (COPC), chronic disease management, pain and palliative care medicine, global health, digital health, community mental health and addiction medicine. FPs with diplomas in mental health, adult medicine, community paediatrics and palliative medicine may be leaders in this focus area, potentially partnering with other specialist colleagues. **‘Acute and district hospital care’**: combined focus on anaesthesia, critical care, and inpatient care. FPs with diplomas in primary emergency care and anaesthesia are best placed to lead this focused hospitalist area. **‘Physical and rehabilitation medicine’:** a previously recognised speciality. Expertise in this field already exists in South Africa. **Additional focus areas** requiring further deliberation to explore and develop consensus: ■ Adolescent health, aesthetic medicine and planetary health ■ There was also interest in developing a focus area in rural and remote health, which may be led by FPs affiliated with the Rural Doctors Association of Southern Africa (RuDASA). Expedition and travel medicine considerations may also be included in this theme.

FP, family physicians; ICU, intensive care unit.

## Discussion

Family medicine’s pluripotential nature and bio-psycho-social approach merge holistic person-centred care with UHC through championing comprehensive PHC. Employing both clinical and non-clinical roles, FPs harness community-wide networks and multiple resources to optimise patient, population and health system outcomes. Participants in this workshop explored how FM subspecialist training can augment much-needed skills and competencies to address the country’s health priorities while protecting the generalist approach at the primary care level. Subspecialist training has the potential to improve care across the PHC platform and expand access to scarce skills, particularly as South Africa’s disease burden overwhelms regional and central facilities, creating bottlenecks to essential services. Most FPs already have specific areas of clinical interest, which are influenced by the local disease burden as well as personal abilities, preferences and aspirations. For example, the intersecting epidemic of communicable and non-communicable diseases in South Africa has resulted in more competent and proficient primary care physicians providing infectious, chronic and palliative care services.^5^

Practical aspects of FM subspecialisation include reviewing the existing CMSA offerings for subspecialisation and expanding entry to include those with FM specialist training. Critical Care, Developmental Paediatrics, Geriatrics, Endocrinology and Rheumatology and perhaps others, lend themselves to such consideration. As for new offerings by the CFP, the multidisciplinary subspecialty of Palliative Medicine is in an advanced stage of development, with approval pending from the Council for Higher Education before the certificate can be offered. Palliative care has also received international attention, with the World Health Organization’s (WHO’s) endorsement indicating its centrality to comprehensive, high-quality and person-centred PHC-oriented healthcare systems.^14,15^ Regarding additional CFP offerings, the Physical Medicine and Rehabilitation is another opportunity under exploration for subspecialisation in FM, as the WHO has acknowledged these services as essential to UHC.^16^

Every context in which FPs find themselves compels the Discipline of FM to imagine a multiverse of practice models. Much of the care required by patients demands a comprehensive, person-centred approach – one that FPs are uniquely positioned to provide. Utilising short to medium-term considerations includes palliative care medicine, which is a good fit for FPs as it cuts across different service domains and requires a generalist approach. This is similar to care related to allergies, geriatrics, addiction, substance use disorders, community health and mental health. Although Rehabilitation Medicine was previously considered a speciality in South Africa, the register closed in the 1980s. However, in 2018, the South African Society of Physical and Rehabilitation Medicine (SASPRM), an affiliate of the International Society of Physical and Rehabilitation Medicine, was formed to revive this field. SASPRM is keen to work with the CFP to resurrect Rehabilitation Medicine, possibly as a subspecialty of FM. The landmark World Health Assembly resolution in 2023 confirmed the need to integrate rehabilitation services into health systems as part of UHC and a strategy for healthy ageing.^17^ Yet, a recent article assessing South Africa’s capacity for rehabilitation services found glaring gaps, indicating an inability to meet the increasing demands. The research highlighted ‘the poor integration of rehabilitation at the primary care level, which has a significant impact on access to rehabilitation’ and expressed concerns about patients obtaining such services if restricted to tertiary institutions.^14,15^

It is essential to clarify that not all subspecialties align well with FM’s principles and practice. Subspecialties that do not discriminate regarding age, gender and personal characteristics would be most suited for FM. Further subspecialisation may also represent pitfalls to be considered at the individual level, the discipline and the broader system. Pitfalls include inadequate exposure to the full scope of the general discipline if subspecialisation occurs too early during postgraduate training and further health system fragmentation if the focus on subspecialisation detracts from the drive to train generalist providers. Prolonged training may also exacerbate student debt and burnout at the practitioner’s level.

Moreover, it is critical to acknowledge the broader discourse on our nation’s Human Resources for Health (HRH) needs.^18^ Key actuarial models^19^ were considered to gauge the supply of and need for medical specialists in South Africa, which informed the draft HRH policy and subspecialist training deliberations by national bodies such as the South African Committee of Medical Deans. Within this regulatory environment, the CFP wishes to engage with key stakeholders such as the CMSA, other speciality disciplines, government, and higher education training institutions to explore future directions, including business models and clarion calls previously considered by other specialities in their quests for subspecialisation.^20^

It is essential to clarify that the intention of this initiative is not to open all existing subspecialties to FPs, but to explore those areas where additional focused training could enhance FM’s contribution to the health system. These can be broadly grouped into three categories:

- Collaborative or multidisciplinary subspecialties, where FPs could function as partners within existing CMSA-recognised subspecialties that share overlapping scopes of practice (e.g. Critical Care, Geriatric Medicine, Endocrinology, Infectious Disease, Rheumatology).
- Emerging multidisciplinary subspecialties, currently under development within South Africa or internationally (e.g. Palliative Medicine and Rehabilitation Medicine), which align closely with the holistic, person-centred philosophy of FM.
- FM-specific subspecialties, envisioned as new domains that build on the discipline’s generalist foundation and respond to contextual service needs (e.g. Rural and Remote Generalism, Maternal and Child Health, Adolescent and Sexual Health, and Community FM).

In most African settings, formal subspecialisation in FM has not yet been established; instead, short courses, modular diplomas and fellowships in areas such as emergency medicine, HIV and palliative care provide limited focused training opportunities. While subspecialisation in FM presents exciting possibilities, it is important to acknowledge several structural and regulatory constraints that will shape its implementation. Under current HPCSA regulations, medical practitioners may register in only one subspecialty after completion of their primary speciality training. This limits the ability of FPs to pursue multiple subspecialist designations and underscores the need for careful prioritisation of areas most aligned with FM’s generalist role.

Training capacity also poses a challenge, as subspecialty posts are limited and usually concentrated at tertiary centres. Expanding such training within FM would require accredited national curricula, funded registrar or fellowship positions and collaboration with existing CMSA structures. Moreover, the district health system – where FPs predominantly function – currently lacks the infrastructure for formal subspecialty rotations or posts, necessitating innovative, decentralised and blended training models.

Finally, given South Africa’s human resource constraints, introducing subspecialisation must not inadvertently deplete the generalist workforce. Instead, it should be designed to strengthen district-level services by enabling FPs with advanced skills to act as consultants, mentors and clinical governance leads within the PHC platform. These considerations will be central to the forthcoming Delphi process, which aims to build a consensus on feasible and contextually appropriate pathways for subspecialisation in FM.

This framework underscores that the purpose of exploring subspecialisation within FM is to strengthen integrated, community-oriented care, and to complement rather than duplicate or compete with existing speciality domains. Clear delineation of these categories may also facilitate collaboration with other colleges and promote alignment with national human resource and service delivery priorities.

## Limitations

The intentions of this workshop were exploratory. Consultation with stakeholders within and outside our discipline, and with health systems leadership, is necessary. We intend to initiate a Delphi process to develop consensus on FM subspecialisation using a more established method. This workshop and its outputs were primarily informed by the perspectives of FM practitioners and registrars, with limited participation from other specialities and institutional stakeholders. As such, the findings reflect a discipline-specific viewpoint and should be interpreted as an initial contribution to a broader national dialogue rather than a definitive framework for implementation. The workshop represented a high-level exploratory exercise, and it did not assess in detail the practical, regulatory or resource implications of subspecialisation. Future work will need to canvass the views of other specialities, universities, the HPCSA, and the Departments of Health, particularly regarding feasibility, funding and the creation of training posts. These steps will be essential to translate the conceptual proposals into actionable plans.

## Conclusion

The scope of practice of FPs is broad. Further training in a relevant subspecialty promotes the acquisition of in-depth skills and knowledge to address priority health problems in South Africa. Expanding the terrain of subspecialisation in FM can strengthen services across the district health system, improve FPs’ job satisfaction and promote FP retention in primary care. While this aspiration will make the discipline more attractive, it will also place FPs on par with their peers globally, where subspecialty training has been implemented. This strategy will also resonate with the discourse in other medical specialities where subspecialisation opens new career directions aligned with contextual needs. However, it is vital that in these endeavours, the generalist FM approach should not be lost.
